# Non-thermal plasma-driven synthesis of Eu^3+^:Y_2_O_3_ nanosized phosphors

**DOI:** 10.1007/s11051-013-2176-2

**Published:** 2013-12-24

**Authors:** Piotr Psuja, Wieslaw Strek, Ihar Yelkin

**Affiliations:** 1 Institute of Low Temperatures and Structure Research, Polish Academy of Sciences, 50-422 Wrocław, Poland; 2 NANTES Ltd. Systems for Nanotechnology, 59-700 Boleslawiec, Poland

**Keywords:** Nanocrystallites, Plasma, Rare earths (RE), Luminescence

## Abstract

The synthesis of nanosized phosphors by using the non-thermal plasma-driven method is presented. The method allows to control the average grain size of nanocrystals. The synthesis of Eu^3+^-doped Y_2_O_3_ nanocrystalline phosphors at water solution of nitrates is described. The average sizes of nanocrystals were controlled by sintering temperature. Their structure, morphology, and luminescent properties were investigated.

## Introduction

The Eu^3+^-doped yttria (Y_2_O_3_) is one of the most popular red phosphors commonly applied in different light-emitting devices (Hunt and Chakhovskoi 1997; Shea 1998). The type of light source determines the specific phosphor requirements. The Eu^3+^:Y_2_O_3_ phosphor is used for wide group of light sources and displays (Hunt and Chakhovskoi 1997; Shea 1998; Shionoya and Yen 1998; Srivastava and Ronda 2003; Vetrone et al. 2004; Wakefield et al. 2000; Waser 2003). There are a few methods for synthesis of nanocrystals (Barta et al. 2010; Chen et al. 2005; Hreniak et al. 2004; Jang et al. 2006; Lee and Choi 2005; Pechini 1967; Tang et al. 2003; Song et al. 2003; Vollath and Szabo 2006; Xu et al. 2005; Zhang et al. 2004). The simple one is modified Pechini method (Pechini 1967; Psuja et al. 2007a, b, 2009, 2012). It consists in crystallization of the compounds in violent combustion reaction. The synthesis connects advantages of sol–gel and thermal decomposition techniques. The disadvantage of this method is the necessity of sintering of resins which implicates the difficulties in the case of mass production. Those difficulties come off as a large amount of air contaminations—products of thermal decomposition of resins. Another drawback of this method is violence of reaction, which really influence and limit mass production application. Despite the above-mentioned methods (Hreniak et al. 2004; Lee and Choi 2005; Pechini 1967; Xu et al. 2005; Song et al. 2003) and the others presented elsewhere (Barta et al. 2010; Chen et al. 2005; Cuba et al. 2010a, b, 2011, 2012; Gbur et al. 2011; Jang et al. 2006; Tang et al. 2003; Vollath and Szabo 2006; Zhang et al. 2004) synthesis of nanocrystals using only water solutions of nitrates exposited for non-thermal plasma (NTP) is possible. The other method of synthesis of nanoparticles uses a plasma microwave reactor (Vollath and Szabo 2006), where the reaction of ionized gases takes place.

In the present work a new method of synthesis of Eu^3+^:Y_2_O_3_ nanocrystals using NTP reactor is described. This method allows fabrication of nanoparticles with narrow size distribution by using only water solutions of metal nitrates. The plasma treatment is necessary to change the crystallization mechanism of salts (nitrates) dispersed in the solution. Such a process of fabrication of nanostructures has been described earlier by (Tereshko et al. 2007a, b).

## Experimental

Lanthanide oxides were purchased form Sigma-Aldrich company. The nitrides were prepared by dissolving the corresponding lanthanide oxides in a nitric acid solution and the water was evaporated from the solution. The structure of obtained powders samples was characterized by X-ray diffraction (XRD-a Stoe Powder Sensitive Detector; filtered CuKα1 radiation). For Eu^3+^:Y_2_O_3_ sample sintered at 600 °C TEM images was created using Philips CM 20 Super Twin Transmission Electron Microscope with resolution 0.025 nm. The photoluminescence spectra were registered at room temperature using CCD spectrophotometer Avantes 350–1,000 nm spectral range, ~0,35 nm resolution. The samples were excited using *λ*_ex_ = 266 nm of Nd:YAG laser system (the forth harmonic of Nd:YAG laser, 10 ns in pulse, 50 Hz). The luminescence spectra of obtained materials were registered and compared.

**Fig. 1:**
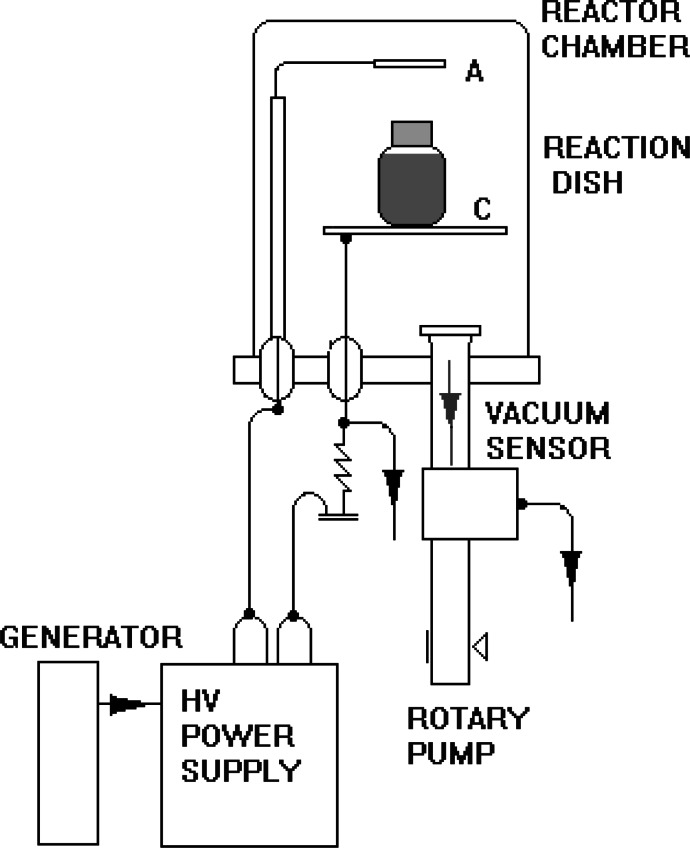
The schematic diagram of NTP reactor

The schematic diagram of NTP reactor is presented above (Fig. 1). The reaction dish contained the water solution of Eu,Y(NO_3_)_3_ was placed between two electrodes. The electrodes were connected to generator, and high-voltage power supply. The reaction dish and electrodes were placed in the vacuum chamber. The pressure in the reactor was decreased to c. a. 0.15 hPa. The voltage and the current were properly 2.5 kV and 30 mA. The process of plasma irradiation takes usually 2–3 h.

### Preparation of Eu^3+^:Y_2_O_3_ nanocrystals

**Fig. 2:**
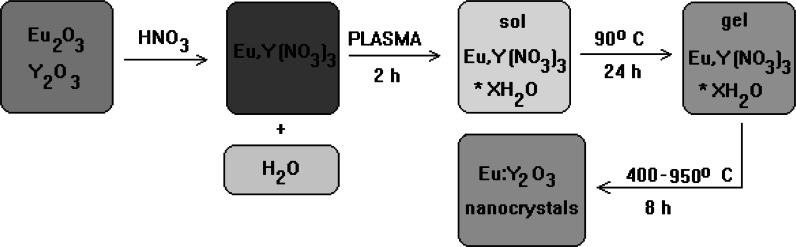
The block scheme of NTP synthesis process of Eu^3+^:Y_2_O_3_

The stoichiometric amounts of yttrium nitride and europium nitride were dissolved using ultrasounds in 10 ml of deionized water. The concentration of europium was established for 5 %. Then the transparent, uniform solution was placed in NTP reactor (Fig. 1). The sample was held in plasma environment at special setup for 2 h. After that solution was divided for 5 samples dried separately at 90 °C and sintered for 8 h at temperatures: 425, 450, 500, 600, 700, 800, 850, and 950 °C. The samples with different Eu^3+^ concentrations (10, 5, 2, 1, and 0.5 %) were also prepared. The scheme of synthesis process is shown in Fig. 2.

## Results and discussion

**Fig. 3:**
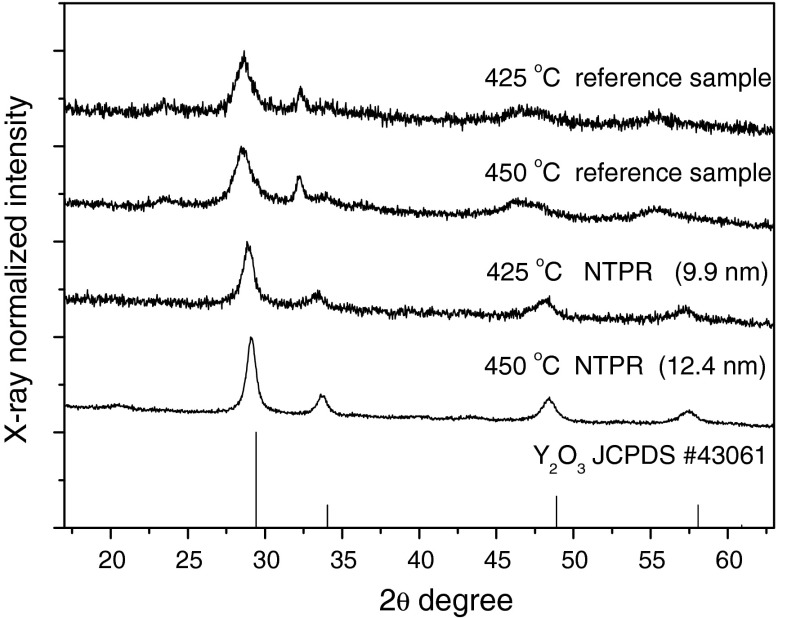
The XRD patterns of Eu^3+^:Y_2_O_3_ synthesized in NTP reactor and the reference sample sintered at the same temperature

**Fig. 4:**
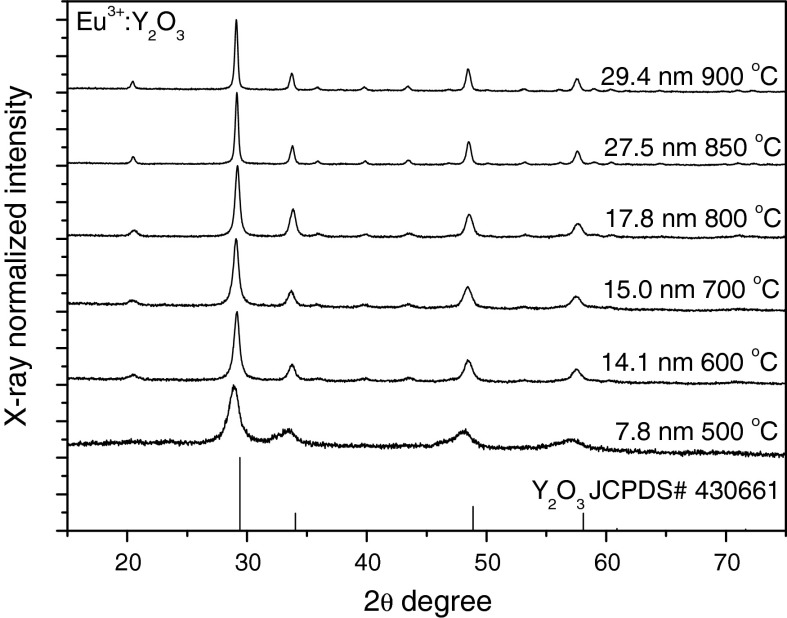
The XRD patterns of Eu:Y_2_O_3_ obtained via non-thermal plasma treatment

**Fig. 5:**
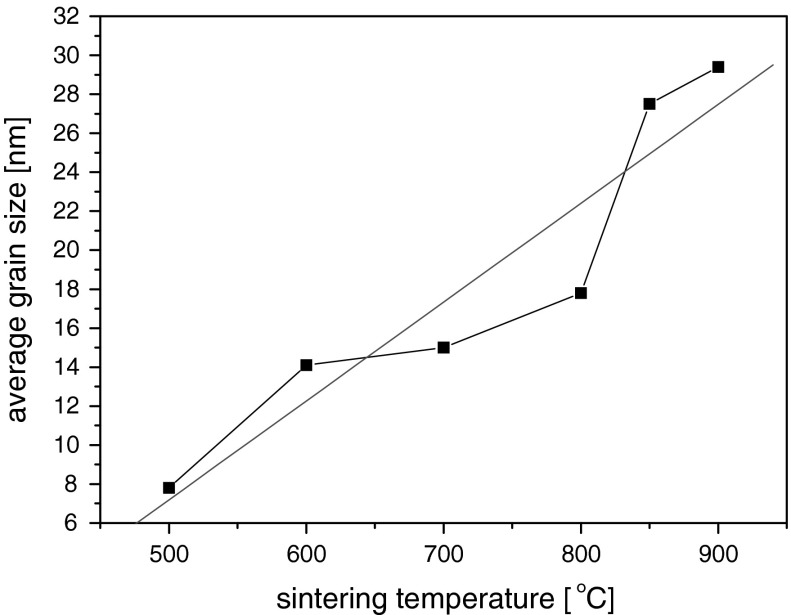
The XRD patterns of Eu^3+^:Y_2_O_3_ nanocrystals with different Eu^3+^ concentrations synthesized by NTP method

The XRD spectra of synthesized materials are presented in Figs. 3, 4, and 5. The XRD curves correspond well with the Y_2_O_3_ pattern (JCPDS# 430661). The average grain sizes of the obtained nanopowders were determined using Scherrer formula from bordering of diffraction peaks.

The solution was divided into two parts. The first was hold in NTP reactor for 3 h and the second was kept as a reference sample. Then the samples were held at temperature 70 °C for 24 h and then sintered at 450 and 425 °C. The XRD curves of samples sintered at 450 and 425 °C were compared. It is seen that sample treated by plasma and sintered at 450 °C was more phase homogenous.

**Fig. 6:**
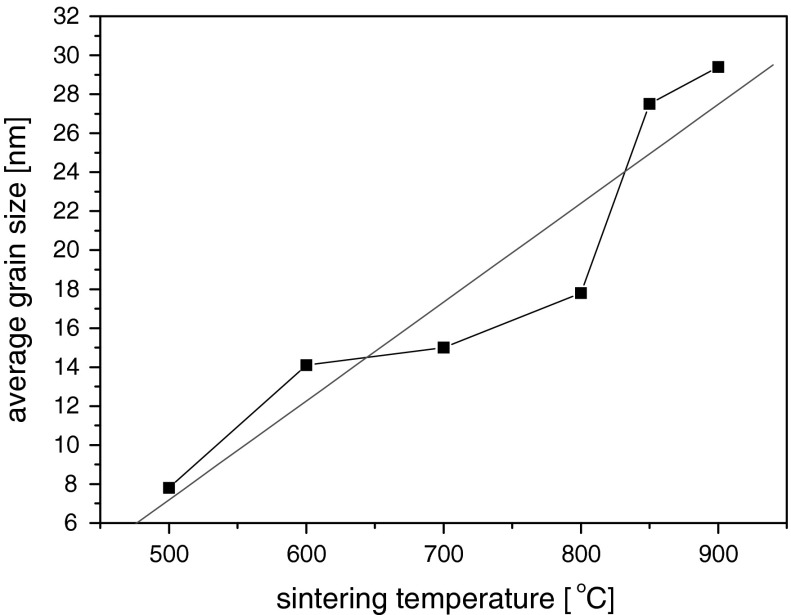
The effect of sintering temperature on the average grain size of Eu^3+^:Y_2_O_3_ nanocrystals synthesized with NTP method

It is seen that with increasing sintering temperature (Figs. 3, 4, and 6) the average grain size increased. This effect allowed to control the size of the obtained nanoparticles by adjusting temperature. The curve for sample synthesized at 500 °C has shown a quite weak crystallization rate. For the samples synthesized at 600, 700, and 800 °C the size differences were very small—14.1 nm at 600 °C and 17.8 nm at 800 °C. However, at 900 °C the average grain size was 29.4 nm. The temperature dependence of average grain size of the obtained nanocrystals at measured temperature range can be approximated as a linear function. It is seen that solution is more temperature sensitive at the range from 800 to 900 °C than from 600 to 800 °C. Hence, the full crystallization of sample appears above 800 °C. The confirmation of this statement is the average grain size of nanocrystals sintered at 850 °C, 27.5 nm. However, as it is shown from TEM image (Fig. 8) of sample sintered at 600 °C the obtained material is crystalline.

The XRD curves of samples sintered at 900 °C with different Eu^3+^ concentrations are presented in Fig. 4. It is seen that average grain size calculated using Scherrer formula decrease with Eu^3+^ concentration.

**Fig. 7:**
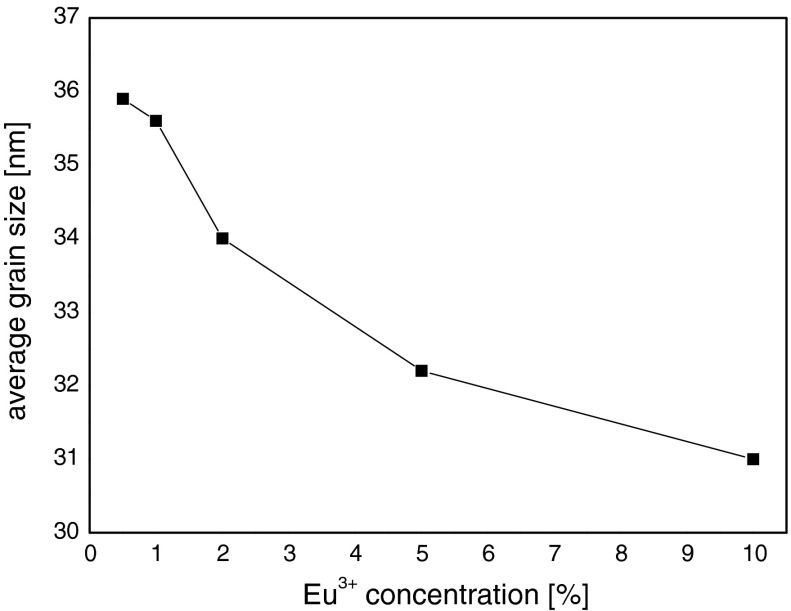
The Eu^3+^ concentration dependence of average grain size of Eu^3+^:Y_2_O_3_ nanocrystals

The dependence of average grain size on Eu^3+^ concentration is shown in Fig. 7. One can see that the average grain size decreases with concentration of Eu^3+^ ions.

Such behavior was also observed earlier for other rare earth ions in Y_2_O_3_, YAG, SnO_2_, and BaTiO_3_ nanocrystals. The most probable explanation is mismatching of ionic and atomic radius of europium and yttrium appearing in strong lattice strength influenced by this difference.

**Fig. 8:**
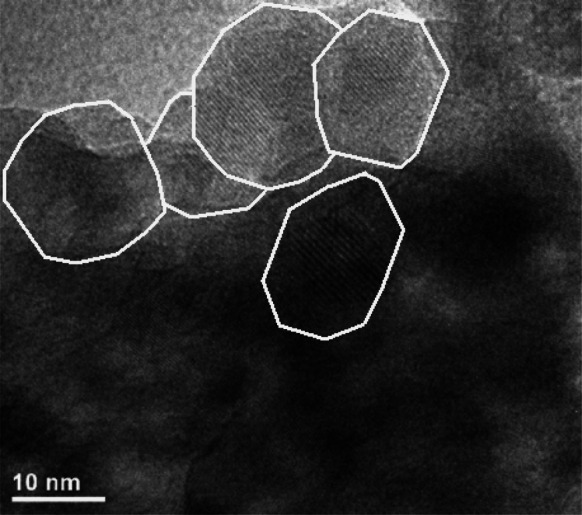
TEM images of Eu^3+^:Y_2_O_3_ obtained using non-thermal plasma reactor, sintered at 600 °C

The TEM images of Eu:Y_2_O_3_ nanocrystals sintered at 600 °C are presented in Fig. 8.

It is seen that size and shape of the obtained nanocrystals is not uniform. The crystals are strongly aggregated. The average grain size estimated from the TEM images is similar to the size calculated from Scherrer’s formula.

**Fig. 9:**
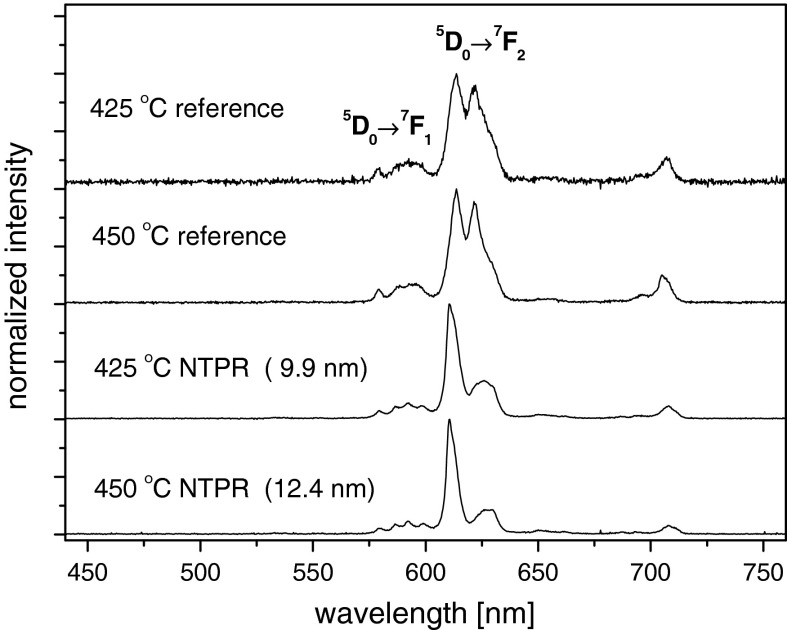
The photoluminescence spectra of Eu^3+^:Y_2_O_3_ sintered at 425 and 450 °C obtained by NTP method and the reference sample

The luminescence spectra of nanocrystals sintered at 425 and 450 °C obtained by using NTP method and the reference one are presented in Fig. 9. It is seen that only samples obtained by plasma treatment showed spectra attributed to Eu^3+^ emission at Y_2_O_3_ host. The spectra of reference samples are typical for Eu^3+^ in amorphous phase. The photoluminescence of the examined materials is well in correlation with their XRD data and leads to conclusion that fabrication of Eu^3+^:Y_2_O_3_ at 450 °C and even at 425 °C in the most simple way from water solution of nitrides is possible only by using NTP reactor.

**Fig. 10:**
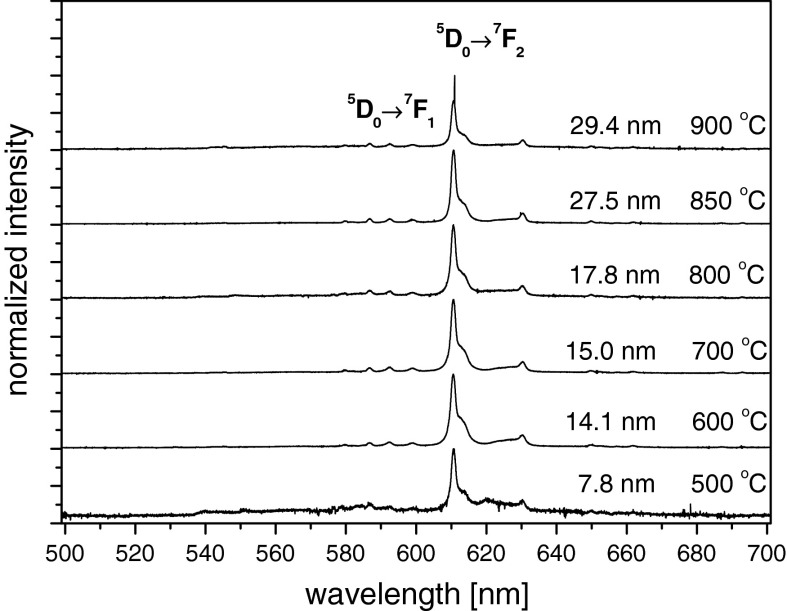
Luminescence spectra of Eu^3+^:Y_2_O_3_ nanocrystals obtained by NTP method with different average sizes of nanocrystals

The photoluminescence spectra under *λ*_exc_ = 266 nm of Eu^3+^:Y_2_O_3_ nanocrystallites with different average grain sizes are presented in Fig. 10. The sizes of samples were controlled using sintering temperature. Any significant differences were observed between spectra sintered above 500 °C. The registered spectra are typical for Eu^3+^ ions in Y_2_O_3_ host. The most intensive are peaks connected with the ^5^ *D*_0_ → ^7^ *F*_2_ transition. The peaks attributed to the ^5^ *D*_0_ → ^7^ *F*_1_ transition are assigned to the magnetic dipole transition and are not affected significantly by the size effect. The second one is electric dipole-allowed transition and is hypersensitive for local crystal field (Morais et al. 2008).

The relative intensities of both the transitions

$$ \beta_{0 \to 1} =  I\left( {{}^{ 5} D_{0} \to{}^{ 7}F_{ 1} } \right)/I_{\text{total}} $$

and

$$ \beta_{0 \to 2} = I\left( {{}^{5} D_{0} \to{}^{ 7}F_{ 2} } \right)/I_{\text{total}} $$

were determined for the samples sintered at different temperatures (see Table 1). Here *I*_total_ is the total intensity of all luminescent transition bands integrated from 450 to 750 nm.

**Table 1:** The *β*_0→1_ and *β*_0→2_ ratios in sintering temperature and average grain size function

Sintering temperature (^°^C)	500	600	700	800	850	900
Average grain size (nm)	7.8	14.1	15.0	17.8	27.5	29.4
*β*_0→1_	0.156	0.133	0.141	0.157	0.133	0.159
*β*_0→2_	0.554	0.256	0.268	0.358	0.267	0.412
*β*_0→2_/*β*_0→1_	3.551	2.015	1.901	2.280	2.008	2.591

**Fig. 11:**
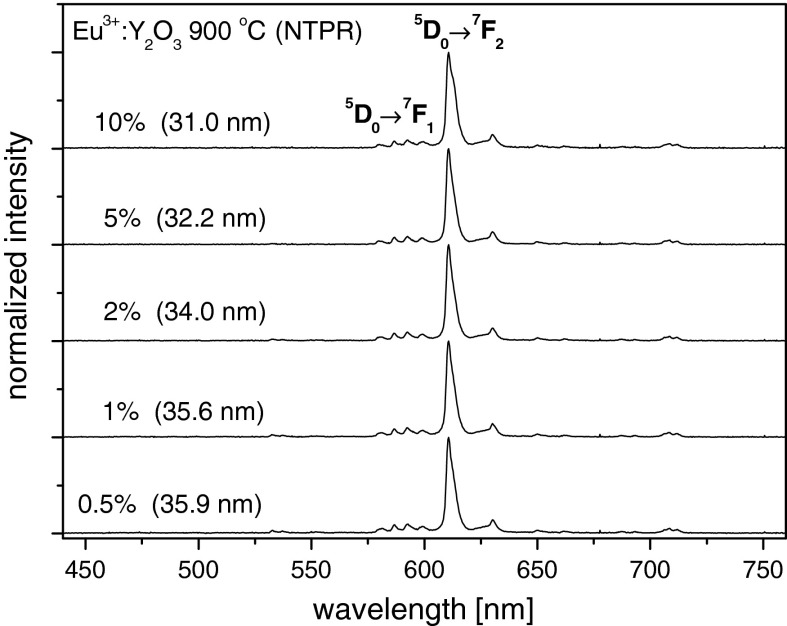
The luminescence spectra of Eu^3+^:Y_2_O_3_ nanocrystals with different Eu^3+^ concentrations obtained by NTP method

**Fig. 12:**
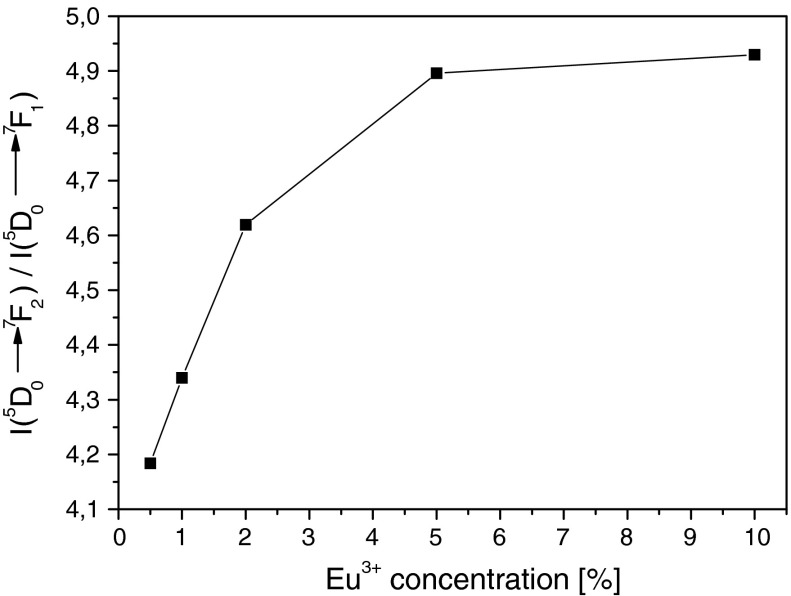
The concentration dependence of *β*_0→2_/*β*_0→1_ ratio of Eu^3+^:Y_2_O_3_ nanocrystals

A different behavior is observed for Eu^3+^ concentration influence on *β*_0→2_/*β*_0→1_. Here, the *β*_0→2_ to *β*_0→1_ ratio increase with concentration (Figs. 11 and 12). The results presented in Fig. 12 are well correlated with the dependence presented in Fig. 7. The observed situation suggests that with increasing of concentration interaction between Eu^3+^ ions and that of crystalline field as in Fig. 7, the size of the obtained nanocrystals decrease with Eu^3+^ concentration. So in single nanograin there is more and more Eu^3+^ ions interacting with local crystalline lattice. However, in the case of 10 % Eu^3+^:Y_2_O_3_ where average grain size of nanocrystals, calculated from Scherrer’s formula is only 31 nm, concentration quenching also plays some role. The same behavior is observed in Fig. 12.

The luminescence studies confirm a good crystallization of Eu^3+^:Y_2_O_3_ crystals obtained by NTP method.

## Conclusion

The Eu^3+^:Y_2_O_3_ nanocrystallites were synthesized by new, simple, and efficient method using NTP reactor. It should be emphasized that only the water solutions of yttrium nitride and europium nitride were used as a substrate of the reaction. The average grain size of the obtained nanocrystals increased with sintering temperature and decreased with Eu^3+^ concentration. It was shown that using NTP reactor the synthesis of efficient Eu^3+^:Y_2_O_3_ is possible even at 425 °C, while reference sample synthesized in the same conditions and even at 450 °C was amorphous to a great extent. The proposed new method allows successful synthesis of RE-doped nanocrystalline phosphors. The resulting Eu^3+^:Y_2_O_3_ nanophophors show sufficient red luminescence upon UV excitation. The examinations over optimal synthesis conditions and size dependence on luminescence features of different nanocrystals will be a subject of our further studies.
